# Highly Selective Transmission Success of Dengue Virus Type 1 Lineages in a Dynamic Virus Population: An Evolutionary and Fitness Perspective

**DOI:** 10.1016/j.isci.2018.07.008

**Published:** 2018-07-18

**Authors:** Carmen Koo, Wei Ping Tien, Helen Xu, Janet Ong, Jayanthi Rajarethinam, Yee Ling Lai, Lee-Ching Ng, Hapuarachchige Chanditha Hapuarachchi

**Affiliations:** 1 Environmental Health Institute, National Environment Agency, 11, Biopolis Way, #06-05-08, Singapore 138667, Singapore; 2 School of Biological Sciences, Nanyang Technological University, 60 Nanyang Drive, Singapore 637551, Singapore

**Keywords:** Disease, Virology, Evolutionary Biology, Phylogenetics

## Abstract

Arbovirus transmission is modulated by host, vector, virus, and environmental factors. Even though viral fitness plays a salient role in host and vector adaptation, the transmission success of individual strains in a heterogeneous population may be stochastic. Our large-scale molecular epidemiological analyses of a dengue virus type 1 population revealed that only a subset of strains (16.7%; n = 6) were able to sustain transmission, despite the population being widely dispersed, dynamic, and heterogeneous. The overall dominance was variable even among the “established” lineages, albeit sharing comparable evolutionary characteristics and replication profiles. These findings indicated that virological parameters alone were unlikely to have a profound effect on the survival of viral lineages, suggesting an important role for non-viral factors in the transmission success of lineages. Our observations, therefore, emphasize the strategic importance of a holistic understanding of vector, human host, and viral factors in the control of vector-borne diseases.

## Highlights

• The sustained transmission of dengue virus 1 lineages is highly selective
• The overall dominance is variable even among the “established” lineages
• The lineage dominance is not merely determined by virus evolution and fitness
• The non-viral factors play an important role in the survival of virus lineages

## Graphical Abstract

**Figure:**
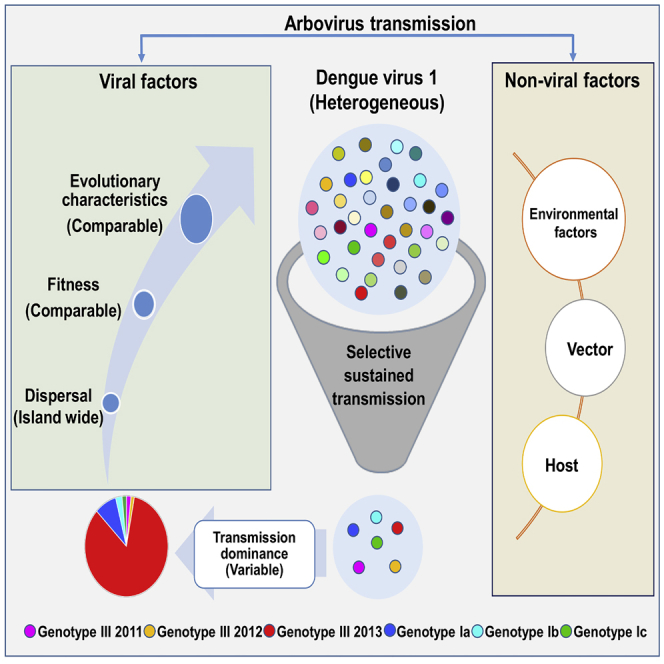

## Introduction

Arthropod-borne viruses (arboviruses) typically survive through horizontal transmission between vertebrate hosts and biting arthropod vectors such as midges, mosquitoes, and ticks. Being RNA viruses, arboviruses demonstrate low fidelity during genome replication (Steinhauer et al., 1992) and exist as diverse populations of closely related variants that collectively behave as “quasispecies” (Domingo et al., 2012). The mutant swarms that constitute “quasispecies” are crucial for virus evolution in dynamic environments. Arboviruses are subjected to evolutionary constraints due to their alternating replication between mammalian and invertebrate hosts (Coffey et al., 2008, Vasilakis et al., 2009). The majority of mutations found in arboviruses, such as Dengue virus (DENV), are deleterious and are eventually weeded out by purifying selection (Holmes, 2003).

DENVs are characterized by extensive genetic diversity and divided into multiple genetically distinct genotypes and lineages (Holmes and Burch, 2000, Holmes and Twiddy, 2003). The genotypes show a characteristic geographical distribution (Holmes and Twiddy, 2003), implying a competitive advantage for individual genotypes in different environments. Rapid evolutionary process of DENV is known to select for strains with enhanced adaptation, generating variants that differ in their ability to spread and cause disease (Twiddy et al., 2002b). The evolutionary dynamics of DENV are influenced by a complex interaction between natural selection and genetic drift, contributing to lineage diversity and dominance (Zhang et al., 2005). The fixation of transient deleterious mutations by stochastic forces is an established phenomenon in DENV populations (Klungthong et al., 2004, Zhang et al., 2005). For instance, seasonal and spatial fluctuations in vector abundance may impose population bottlenecks on endemic strains, thus allowing new viruses to establish in an ecological niche (Scott et al., 2000). In addition, cross-protective immunity among DENV serotypes plays an important role in governing the transmission potential of circulating strains in a particular locality (Adams et al., 2006, Chen and Vasilakis, 2011). The differential susceptibility among new viruses against cross-reactive immune responses elicited by preceding serotypes may give rise to serologic-escape mutants that sustain transmission and cause cyclical epidemics (Adams et al., 2006). These observations suggest that the ability of DENV strains to establish transmission in a given locality is influenced by the epidemiological landscape shaped by a combination of viral, immunological, vector, and environmental determinants (Lourenco and Recker, 2010).

DENV is hyper-endemic in Singapore, where all four serotypes circulate (Lee et al., 2012), with DENV-1 and DENV-2 being the most common. One serotype typically dominates at any given time. The switching of dominant serotypes has been associated with epidemics (Hapuarachchi et al., 2016b, Lee et al., 2010). There have been two large epidemics due to DENV-1 in the country in 2005 (Koh et al., 2008) and 2013–14 (Hapuarachchi et al., 2016a, Hapuarachchi et al., 2016b). The dominant genotypes during each epidemic were different; genotype I in 2005 (Schreiber et al., 2009) and genotype III in 2013–14 (Hapuarachchi et al., 2016a, Hapuarachchi et al., 2016b). In addition, the laboratory-based virus surveillance has identified a plethora of less-dominant, yet genetically distinct strains since 2008. Virus surveillance plays an important role in the integrated vector control program in Singapore by providing regular updates on the circulating virus strains, which helps to assess the epidemic risk of any newly emerging virus, and thereby to prioritize control efforts on the ground. Being a vibrant trade and travel hub in a dengue hyper-endemic region, the introduction of new viruses and the rapid evolution of circulating viral lineages sustain the high diversity of DENV in Singapore (Lee et al., 2012). This provides many opportunities to explore the evolutionary processes among DENVs that go through bottlenecks due to dynamic fluctuations of immunological pressure and vector population density.

In the present study, we conducted a molecular epidemiological analysis of DENV-1 genotypes collected island wide between 2011 and 2016, using both envelope (*E*) gene and complete genome sequences. Our aim was to understand the origin, evolution, dispersal, and transmission success of different lineages of DENV-1 circulated during the study period. Considering viral replication in host cells as a surrogate for the fitness, we demonstrate that the variable transmission success of lineages that established sustained transmission is shaped by stochastic forces, which are likely to be influenced by non-viral factors more than the viral fitness and evolutionary differences.

## Results and Discussion

Among 18,696 individuals tested between January 2011 and December 2016, 4,252 patients (22.7%) were positive for DENV NS1 antigen. DENV serotypes were determined in 3,946 (92.8%) NS1-positive sera. DENV-1 (56.7%) was the dominant serotype, followed by DENV-2 (33.5%), DENV-3 (7.6%), DENV-4 (2.1%), and mixed serotypes (0.1%; DENV-1 and DENV-2). Of them, 3,363 samples of all serotypes were genotyped by using *E* gene sequences. The present study reports the analysis of only DENV-1, which includes 1,963 *E* genes and 239 complete polyprotein sequences.

### DENV-1 Population was Highly Heterogeneous, but Only Six Lineages Established Sustained Transmission

*E* gene sequences were used to determine the weekly dynamics of DENV-1 heterogeneity. The *E* gene collection included 29 sequences reported in 2011, 49 in 2012, 728 in 2013, 990 in 2014, 93 in 2015, and 74 in 2016. Whole genome sequencing was performed on a subset of samples selected to represent different groups of viruses identified based on *E* gene phylogeny to understand virus evolution during the study period. In addition, we completed the polyprotein sequences of two genotype III isolates detected in 2008 and 2009 for comparison purposes.

**Figure 1:**
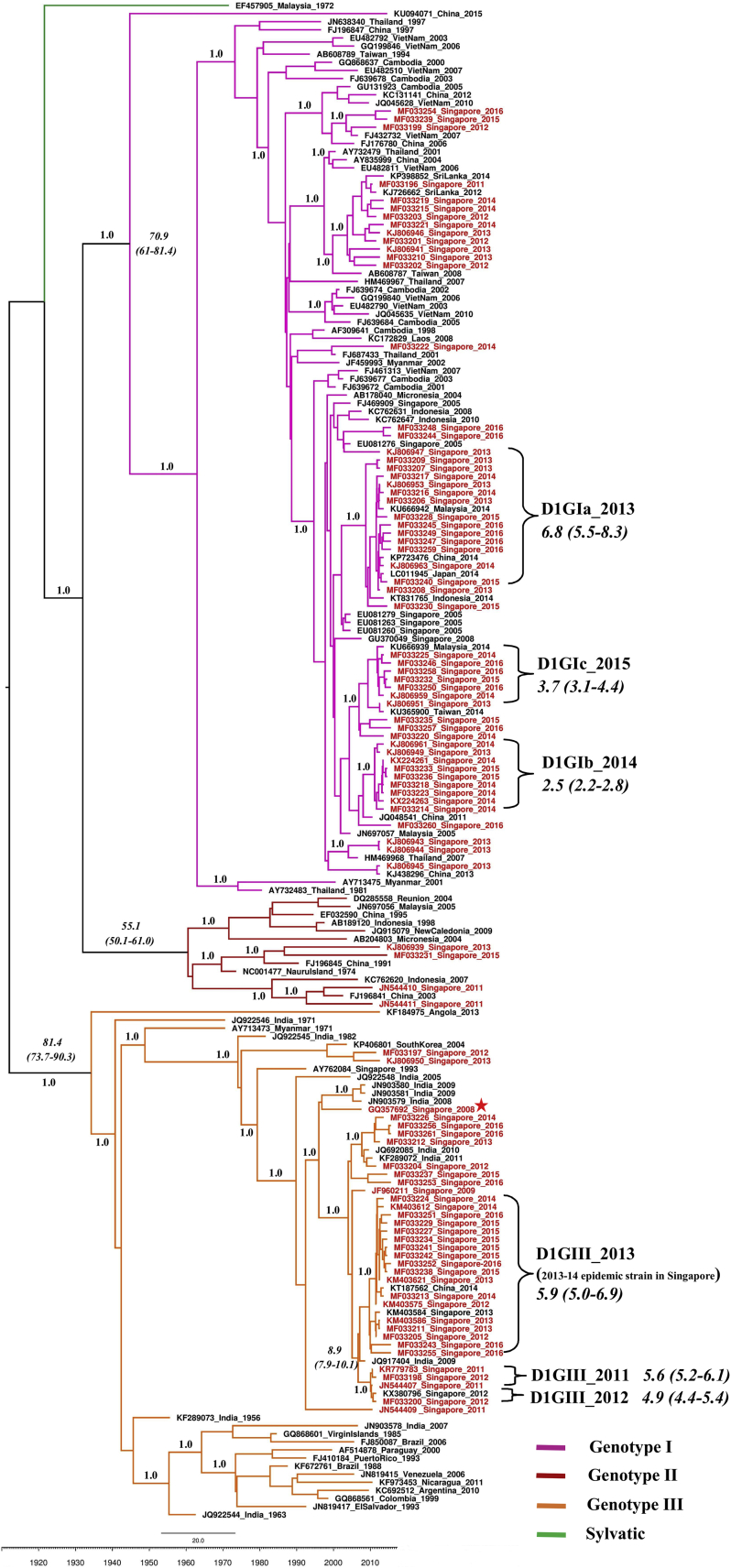
Phylogenetic and tMRCA Analysis of DENV-1 The time-scaled maximum clade credibility tree was constructed using the Bayesian Markov Chain Monte Carlo (MCMC) method implemented in the BEAST package v1.7.4. The analysis included 93 complete polyprotein sequences selected to cover all possible viral genetic diversity observed during this study (highlighted in red) and 94 sequences retrieved from GenBank database. Each genotype is shown in different colors as per the tree legend. The local strain (imported case from India) that clustered in the outgroup of genotype III lineages is shown with an asterisk. Numbers on branches represent the posterior probability values. The tMRCA values in years are shown in italics, with 95% highest posterior density (HPD) values in brackets. The scale bar shown above the time scale is substitutions/site/year.

**Figure 2:**
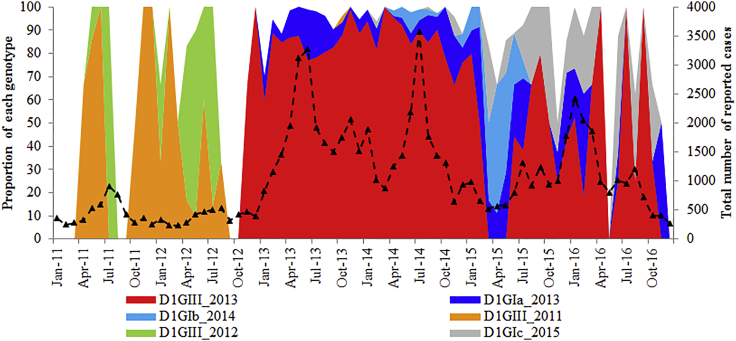
Time of Emergence, Extinction and Temporal Fluctuations of Each “Established” Lineage from 2011 to 2016 The figure was generated based on the genotype information gathered from the analysis of 1,963 complete *E* gene sequences. The longitudinal pattern of total reported cases is shown in a broken line to demonstrate the varying dominance of six “established” lineages during periods of different transmission intensity. D1, DENV-1; GI, genotype I; GIII, genotype III.

There were 36 genetically distinct DENV-1 strains of multiple lineages (please see the Transparent Methods section for the definition of a strain). Notably, only six (16.7%) of them (genotype III 2011, genotype III 2012, genotype III 2013, genotype Ia, genotype Ib, and genotype Ic) established sustained transmission and formed distinct lineages with strong posterior probability support (Figure 1). Their mean evolutionary rates were comparable (Tables 1 and 2) and consistent with the estimated rates obtained for DENV-1 in previous reports (Sun and Meng, 2013, Twiddy et al., 2003). Each “established” lineage shared a common regional ancestor (Figure 1; Tables 1 and 2), indicating the cross-border virus sharing. The origin and divergence data analyses (Tables 1 and 2) revealed that the emergence period of “established” lineages ranged from 2008 to 2014 and the median age of each lineage did not differ substantially, suggesting that different virus lineages emerged in a parallel timescale and co-circulated subsequently. Genotype III 2013 was the dominant lineage during the 2013–14 epidemic (Hapuarachchi et al., 2016a, Hapuarachchi et al., 2016b). Figure 2 shows the time of emergence, extinction, and temporal fluctuations of each “established” lineage from 2011 to 2016.

**Table 1:** Summary of Evolutionary Analysis of “Established” Lineages of DENV-1 Genotype III

DENV-1 Strains	Genome-wide Genetic Signature (Non-synonymous)	Mean Evolutionary Rate^a^ (95% HPD)	tMRCA^b^ (95% HPD)	Closest Ancestor (Posterior Probability)
GIII 2011	prM-I120M + E-I129T + E-F337I + NS1-D278N	7.3 (2.8–12.7)	5.6 (5.2–6.1)	India 2009 (0.997)
GIII 2012	prM-I120M + E-I129T + E-S449N + NS2A-S142P	8.1 (3.9–14.0)	4.9 (4.4–5.4)	India 2009 (0.997)
GIII 2013	E-F337I + NS1-D278N	5.5 (2.8–8.5)	5.9 (5.0–6.9)	India 2009 (0.976)

GIII, genotype III; HPD, highest posterior density; tMRCA, time to most recent common ancestor.

a Expressed in ×10^−4^ nucleotide substitutions/site/year.

b Expressed in years, to be calculated from 2016.

**Table 2:** Summary of Evolutionary Analysis of “Established” Lineages of DENV-1 Genotype I

DENV-1 Strains	Genome-wide Genetic Signature (Non-synonymous)	Mean Evolutionary Rate^a^ (95% HPD)	tMRCA^b^ (95% HPD)	Closest Ancestor (Posterior Probability)
GIa	NS1-F323Y + NS2A-M168I + NS5-I114V + NS5-V231I + NS5-N285C + NS5-P525S + NS5-D554E + NS5-L562Q	6.8 (4.6–9.6)	6.8 (5.5–8.3)	Singapore 2005 (0.998)
GIb	preM-V135A + E-S338L + E-T359S + NS2B-L21F + NS4A-V68M + NS5-P137H + NS5-I181V + NS5-V413I + NS5-P525S	8.0 (3.8–13.2)	2.5 (2.2–2.8)	China 2011 (0.998)
GIc	NS2B-I106 + NS5-V413I	7.7 (3.8–12.3)	3.7 (3.1–4.4)	Singapore 2013 (0.998)

GI, genotype I; HPD, highest posterior density; tMRCA, time to most recent common ancestor.

a Expressed in ×10^−4^ nucleotide substitutions/site/year.

b Expressed in years, to be calculated from 2016.

### DENV-1 Strains were Widely Dispersed and Showed Distant Diffusion Pathways

**Figure 3:**
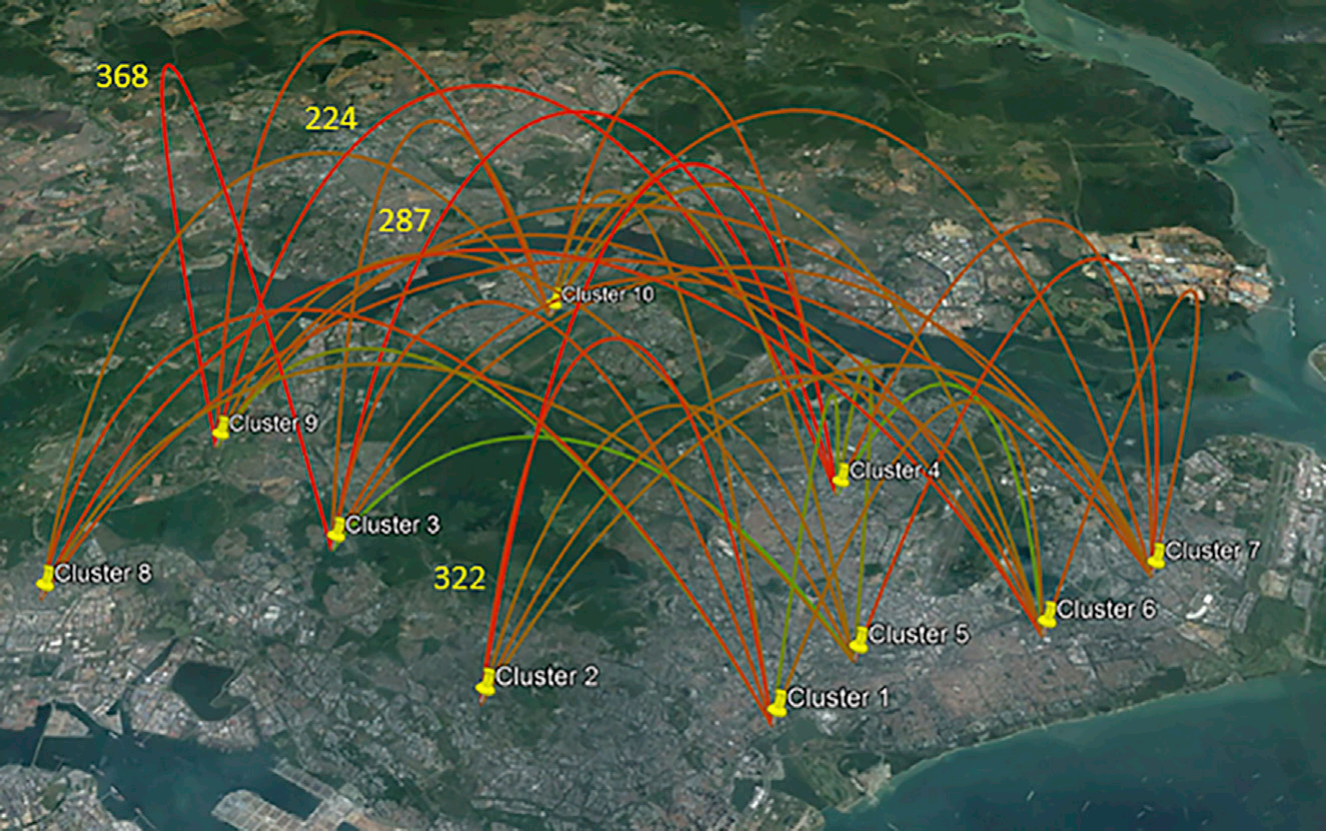
Dispersal Pattern of DENV-1 Population in Singapore from 2011 to 2016 The dataset included 792 complete *E* gene sequences of local DENV-1 isolates, and the sampling locations were classified into 10 clusters; cluster 1, Toa Payoh/Beach Road/Kim Keat Road; cluster 2, Clementi/Leedon Heights; cluster 3, Bukit Batok/Bukit Timah; cluster 4, Ang Mo Kio/Serangoon/Hougang; cluster 5, Geylang; cluster 6, Kaki Bukit/Ubi/Telok Kurau; cluster 7, Tampines; cluster 8, Jurong West/East, cluster 9, Choa Chu Kang/Sungei Kadut; cluster 10, Yishun/Woodlands. The figure shows 35 well-supported epidemiological links (Bayes factor, BF > 3). The branches are colored based on the BF values; red, orange, and green indicate high, intermediate, and low values, respectively. The highest BF values are shown in numbers. See also Figure S1; Tables S1 and S2.

In contrast to other regional countries (Raghwani et al., 2011), our phylogeography analysis revealed a weak spatial clustering of DENV-1 population in Singapore, implying its widespread and complex distribution in local settings (please see the Transparent Methods section for the assigning of location information to sequences). The analysis revealed 35 well-supported (Bayes factor [BF] > 3) diffusion pathways (Figure 3; see Table S1). Of them, 13 pathways demonstrated decisive support (BF > 100). The highest connectivity was observed in clusters 4 (Ang Mo Kio/Serangoon/Hougang), 6 (Kaki Bukit/Ubi/Telok Kurau), 7 (Tampines), and 10 (Yishun/Woodlands), suggesting an important role for these areas in DENV-1 dissemination in the country. The most probable ancestral locations of six “established” strains were in South Central Singapore (Figure 3; see Table S2), which is historically a dengue hot spot. More importantly, among 13 diffusion pathways with BF > 100, 9 (69.2%) were distant links (West-East) (Figure 3). Being a small island, the well-developed transportation network and a highly mobile population could have facilitated the virus spread between distant locations in Singapore. The estimated root state posterior probability was highest in clusters 9 (0.106) and 10 (0.104), followed by clusters 4 (0.103) and 2 (0.103), indicating that they were the major sources of DENV-1 diversity in Singapore (see Figure S1). Clusters 9 and 10 are in close proximity to (approximately 5–10 km) the Woodlands Checkpoint, which is one of the immigration checkpoints, suggesting the potential of virus sharing across the northern border between Singapore and Malaysia (Ng et al., 2015).

### “Established” Lineages were Introduced Independently and Evolved Further *In Situ*

**Figure 4:**
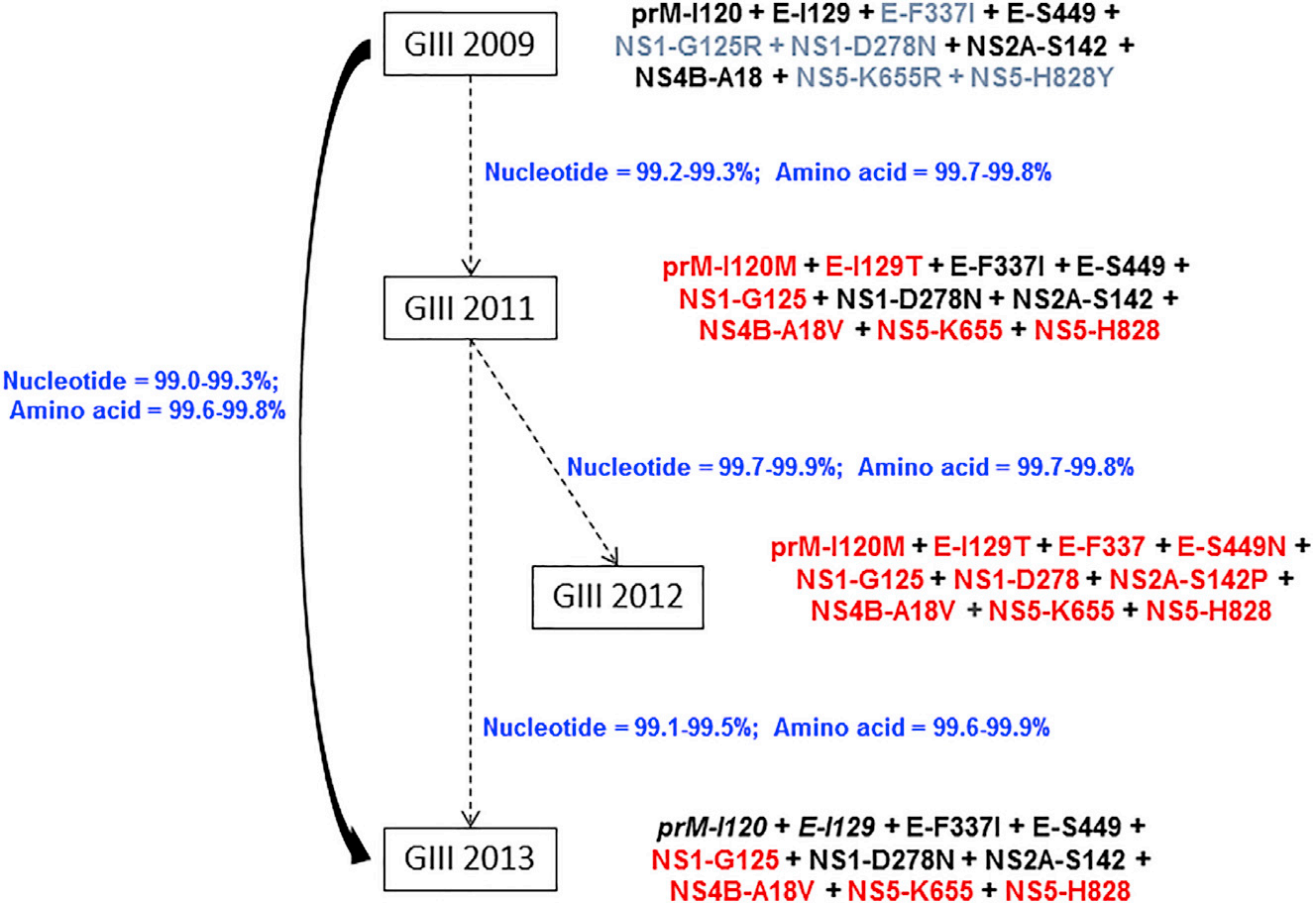
Evolutionary Characteristics of DENV-1 Genotype III Lineages that Established Transmission from 2011 to 2016 The analysis included 144 complete genome sequences of genotype III collected between 2009 and 2016. The isolate in 2009 [SG(EHI)D1/0091Y09] was detected in a sporadic case. It was ancestral to all three “established” genotype III lineages. The 10-amino acid signature is shown next to each lineage/strain, amino acid substitutions in red and reverse mutations in italics. The percentage of nucleotide and amino acid sequence identity is indicated in blue. GIII, genotype III.

Until the emergence of genotype III 2011 in April (E-week 14) 2011 (Figure 2), our surveillance has captured only two DENV-1 genotype III strains reported locally in 2008 and 2009. Whole genomes of both virus strains were included in the current analysis. To decipher the genetic relationship among DENV-1 genotype III lineages reported from 2009 to 2014, we identified a genome-wide 10-amino acid signature that distinguished each lineage (Figure 4). Despite having the highest genetic similarity (99.2%–99.3% of nucleotide and 99.7%–99.8% of amino acid) with the 2009 isolate, genotype III 2011 lineage (n = 9) (prM-I120M, E-I129T, and NS4B-A18V) was distinguishable from the 2009 isolate (NS1-G125R, NS5-K655R, and NS5-H828Y) by six amino acid substitutions (Figure 4). Even though the circulation of genotype III lineages was overlapping and successive (Figure 2), mutation patterns showed a unique mixture of forward and reverse substitutions. For an example, prM-I120M and E-I129T substitutions found in genotype III 2011 were wild-type in the genotype III 2013 epidemic lineage. Likewise, E-F337I and NS1-D278N substitutions found in both 2011 and 2013 lineages were wild-type in genotype III 2012 isolates (Figure 4).

This swapping mutation identity lacked a temporal direction and indicated a mixed (forward-reverse) evolutionary pattern. This was further supported by the time to most recent common ancestor (tMRCA) analysis that showed a longer period of emergence (median 5.9 years from 2016; 95% highest posterior density [HPD] 5.0–6.9 years) for the genotype III 2013 epidemic lineage than the 2012 lineage (median 4.9 years from 2016; 95% HPD 4.4–5.4 years). Given the relatively short time gap of emergence between each lineage, limited number of genotype III strains detected during 2009–12, and lack of intermediate sequences, we postulated that DENV-1 genotype III lineages observed since 2011 have been introduced rather than emerging through an *in situ* evolutionary process.

Phylogenetic analysis of whole-genome sequences revealed that DENV-1 sequences reported during 2008–09 from India (GenBank: JN903580, JN903581, and JN903579) formed the outgroup of local genotype III lineages (Figure 1). Another locally reported virus strain (GenBank: GQ357692) that clustered with the “outgroup” sequences (Figure 1) was obtained from an imported case from southern India. Indian sub-continent strains and local genotype III lineages shared a unique genetic signature of C-L46M + NS5-L123I substitutions. Of the two substitutions, NS5-L123I is unique and novel among DENV-1 genotype III sequences known to date. These findings suggested that local genotype III lineages were ancestral to Indian sub-continent isolates. Based on C-prM region sequences from the GenBank database, it was evident that isolates with C-L46M substitution have circulated in northern India in 2010 (GenBank: JF276796) and 2012 (GenBank: KC787088). However, DENV-1 genotype III sequences reported in and before 2006 did not possess the C-L46M substitution (Kukreti et al., 2008, Patil et al., 2011). This observation is in agreement with our tMRCA analysis that suggested the emergence of local genotype III lineages to be between 2006 and 2008 (median 8.9 years from 2016; 95% HPD 7.9–10.1 years).

Another notable genetic link between the Indian sub-continent strains and local genotype III lineages was a 21-nucleotide deletion (10,296–10,316; GenBank: NC001477) in the hypervariable region (HVR) of the 3′ UTR. The deletion was present in all DENV-1 genotype III sequences that possess C-L46M and NS5-L123I substitutions (see Figure S2). Earlier studies suggest that this 3′ UTR deletion is a unique marker of DENV-1 circulating in the Indian sub-continent from 2006 onward (Dash et al., 2015). Together with our findings, it is evident that a new lineage of DENV-1 genotype III that possessed C-L46M + NS5-L123I substitutions and the 21-nucleotide deletion in 3′ UTR has emerged in the region around 2006.

Among three genotype I lineages (denoted as genotype Ia, Ib, and Ic) that established sustained transmission, genotype Ia was the most common and contributed to the second highest case burden during the 2013–14 epidemic (Hapuarachchi et al., 2016b). Although each genotype I lineage possessed a distinct amino acid signature (Table 2), all three lineages shared several similar features. They were newly emerged viruses circulating in the region at a similar timescale. The tMRCA analysis indicated a recent ancestry for genotypes Ib and Ic (Table 2), approximately at the same time of their emergence in Singapore. All lineages, except genotype Ib, included isolates reported from regional countries during the same period (Figure 1), indicating their widespread presence in Southeast Asia and the possibility of them being introduced into Singapore.

**Figure 5:**
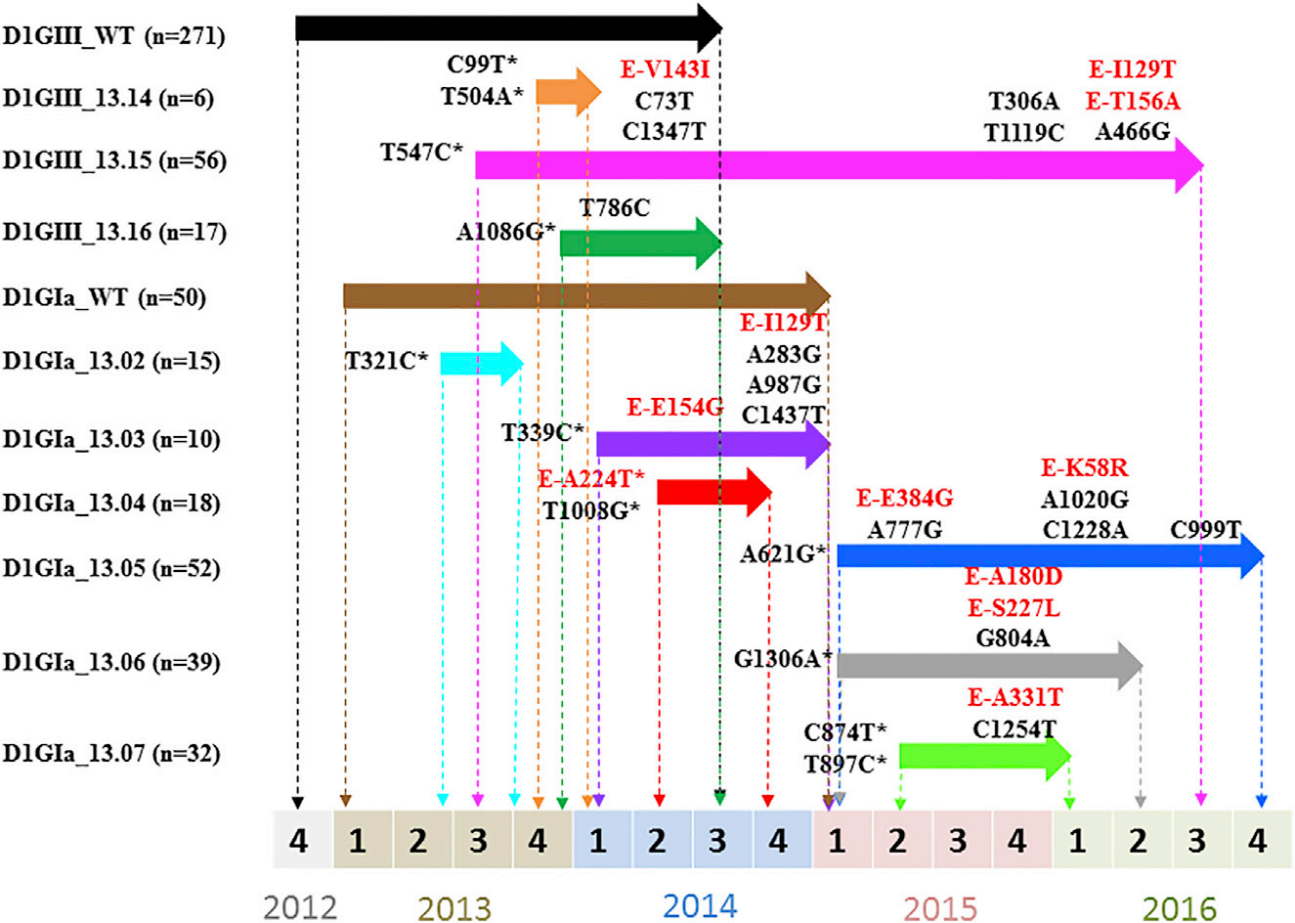
Mutation Profiles of Variants of DENV-1 Genotype III 2013 and Genotype Ia Lineages The figure illustrates temporal pattern of emergence, maintenance, and extinction of each variant during the study period. The classification of DENV-1 genotype III variants (variant 13.01–13.13) has been described in details elsewhere (Hapuarachchi et al., 2016a). Arrows indicate the transmission period of each variant. A timeline is given below the figure with numbers 1–4 to represent each quarter of the year (1: Jan–Mar; 2: Apr–Jun; 3: Jul–Sep; 4: Oct–Dec). Substitutions shown are the *E*-gene-based signature mutations of each variant. Initial substitutions were considered as founder substitutions and have been shown with an asterisk. Those that appeared later in respective variants were named as “secondary” substitutions (without an asterisk) in the text. Those shown in red are “fixed” non-synonymous substitutions. Remaining residues are “fixed” synonymous substitutions. Positions of all amino acid substitutions are shown at the *E* gene level, whereas the synonymous substitutions are numbered from the beginning of the polyprotein. D1GIII, genotype III 2013 lineage; D1GIa, genotype Ia lineage; n, number of sequences belonging to each variant, WT, wild-type.

Besides introductions, *in situ* evolution also played an important role in driving the micro-scale diversity of “established” lineages. For example, the detailed analysis of 1,582 complete *E* gene sequences of the genotype III 2013 lineage demonstrated a consistent micro (*in situ*) evolutionary process that is likely to be facilitated by the intense transmission during the epidemic period (Hapuarachchi et al., 2016a). The analysis identified 15 genetically distinguishable variants (wild-type and additional 14 variants) based on fixed nucleotide substitutions in *E* gene (Hapuarachchi et al., 2016a). Of them, the evolutionary dynamics of 12 variants have been described in detail elsewhere (Hapuarachchi et al., 2016a). Among the remaining three variants (13.14–13.16), variant 13.16 (n = 17) was the most common group, followed by variants 13.15 (n = 10) and 13.14 (n = 6). Similarly, genotype Ia was also composed of multiple variants. Each variant possessed characteristic founder and non-founder substitutions that distinguished one another (Figure 5).

### “Established” Lineages Shared Similar Evolutionary Characteristics, but were Not Equally Dominant

Despite the widespread distribution in the region, there was limited evidence of positive selection in “established” lineages. In general, they were either under purifying selection or evolutionarily neutral in our analysis (Tables 3 and S3). This finding is consistent with the notion that purifying selection generally drives the evolution of DENV (Holmes and Twiddy, 2003, Teoh et al., 2013, Twiddy et al., 2002a, Twiddy et al., 2002b, Vasilakis and Weaver, 2008). Unlike other RNA viruses, the evolution of arboviruses is constrained by dual replication in mammalian and invertebrate hosts, producing negative fitness trade-offs among traits favored in each host (Deardorff et al., 2011, Novella et al., 1999, Vasilakis et al., 2009). Recent studies have also revealed that DENV intra-host genetic diversity in mosquitoes is largely shaped by genetic drift and purifying selection (Lequime et al., 2016, Lequime et al., 2017). The only exception was NS5-N285C substitution uniquely detected in genotype Ia, which was predicted to be under positive selection by fixed effects likelihood (FEL), internal fixed effects likelihood (IFEL), and mixed effect model of evolution (MEME) methods (Table 3). NS5-285 resides within the RNA-dependent RNA polymerase catalytic domain of NS5 (Klema et al., 2016). However, further studies are needed to determine whether NS5-N285C substitution provided any adaptive advantage to genotype Ia. Interestingly, the mean evolutionary rates among “established” lineages were also comparable (Tables 1 and 2). These observations suggested a role for genetic drift in the apparent “selection” of lineages that established sustained transmission.

**Table 3:** Selection Pressure Analysis on Genome-wide Non-synonymous Substitutions Detected in Six “Established” Lineages of DENV-1

Substitution^a^	Lineage	Selection Pressure Analysis^b^				
		SLAC	FEL	IFEL	FUBAR	MEME
E-I337F	GIII 2011; GIII 2013	Neg (0.01)	Neg (0.004)	Neu	Neg (0.99)	Epi (0.04)
E-S338L	GIb	Neu	Neg (0.04)	Neg (0.05)	Neg (0.93)	Neu
NS1-F323Y	GIa	Neg (0.04)	Neg (0.02)	Neu	Neg (0.99)	Neu
NS2B-L21F	GIb	Neg (0.001)	Neg (<0.001)	Neg (0.04)	Neg (0.99)	Neu
NS2B-I106	GIc	Neg (0.02)	Neg (0.03)	Neu	Neg (0.97)	Neu
NS4A-V68M	GIb	Neg (0.04)	Neg (0.01)	Neu	Neg (0.99)	Neu
NS5-V231I	GIa	Neg (0.04)	Neg (0.01)	Neu	Neg (0.99)	Neu
**NS5-N285C**	**GIa**	**Neu**	**Pos (0.03)**	**Pos (0.01)**	**Neu**	**Epi (0.002)**

a NCBI reference genome NC_001477 was used as the wild type sequence.

b The data are given for the residues identified in genetic signatures of each “established” lineage (Tables 1 and 2). The table includes only the sites that were either under purifying or positive (shown in bold) by at least three methods. The neutral sites are given in Table S3. p values (SLAC, FEL, IFEL, and MEME) and posterior probability (FUBAR) are given in brackets. E, envelope protein; Epi, episodic selection; GI, genotype I; GIII, genotype III; prM, precursor membrane protein; Neg, purifying selection; Neu, neutral; NS, non-structural proteins; Pos, positive selection.

Even though genotype I lineages demonstrated the ability to establish sustained transmission, the proportions of genotype Ia (8.2%), Ib (2.4%), and Ic (1.7%) were much lower than that of genotype III 2013 epidemic (81.3%) lineage. This was despite the intense transmission conditions that prevailed during the time of their emergence (Figure 2). The sustainability of virus transmission depends on the availability of a susceptible human pool and a vector population. Unlike in humans, flaviviruses cause a long-lived and persistent infection in vector mosquitoes (Salas-Benito and De Nova-Ocampo, 2015). The adult female mosquitoes have a lifespan of 1–3 weeks depending on the environmental and non-environmental conditions (Christofferson and Mores, 2016, Liu-Helmersson et al., 2014, Maciel-de-Freitas et al., 2007) such as temperature (Goindin et al., 2015, Maciel-de-Freitas et al., 2007), daily temperature fluctuations (Lambrechts et al., 2011), and virus infection (Christofferson and Mores, 2016). As the virus utilizes host cell pathways and resources to replicate, it is unlikely for another similar or closely related virus strain to infect the same mosquito in a subsequent infection, according to the phenomenon of superinfection (homologous) exclusion (Condreay and Brown, 1986). The co-infection, and especially super-infection (Pepin et al., 2008), may result in competition for host resources (Read and Taylor, 2001) and shape the pathogen virulence and transmissibility, thereby affecting its population dynamics (May and Nowak, 1995, Mosquera and Adler, 1998, Nowak and May, 1994). It has previously been shown that C6/36 *Aedes albopictus* cells infected with four DENV serotypes are resistant to heterotypic super-infection (Dittmar et al., 1982). This suggests that when a particular DENV lineage is dominantly transmitted, there is a weak opportunity for lineages introduced subsequently to establish and spread, especially when *Aedes* mosquito population is consistently suppressed. Our observations on genotype I lineages support this notion. For example, genotype Ic was apparent in large clusters in early 2016, although the lineage was first identified in Singapore in February 2014 (Figure 2). Moreover, 57.4% (27/47) of genotype Ib isolates were detected in clustered cases and no other “established” lineages were detected in the majority (76.5%) of those dengue clusters, indicating the highly localized nature of genotype Ib transmission. In Singapore, the *Aedes aegypti* population is controlled through a multi-pronged approach (Hapuarachchi et al., 2016b), and this may explain why lineages other than the epidemic lineage struggled to dominate.

### *In Vitro* Replication Kinetics Suggested that Variable Transmission Success of “Established” Lineages Is Unlikely to Be due to Increased Fitness

To determine whether the improved fitness contributed to the variable transmission success observed among “established” lineages, we compared the *in vitro* replication kinetics of six lineages in mammalian (Huh-7) and mosquito (C6/36) cell lines. We also included two genotype I strains (D1/SG/05K4441DK1/2005 and D1/SG/05K4443DK1/2005) dominant during the epidemic in 2005, the strain [SG(EHI)D1/0091Y09] that was ancestral to all three genotype III lineages (Figure 1), and SG(EHI)D1/09106Y11, an isolate that did not possess the 21-nucleotide deletion in 3′ UTR observed in the genotype III lineages for comparison purposes. SG(EHI)D1/09106Y11 was detected in a sporadic, single case in 2011. All strains showed similar replication profiles in C6/36 *Aedes albopictus* cell line, although the peak titers were variable and strain dependent, but were not significantly different among strains. This observation agrees with previous reports (Tajima et al., 2007), suggesting that vector adaptation is unlikely to explain the variable transmission success of six lineages.

**Figure 6:**
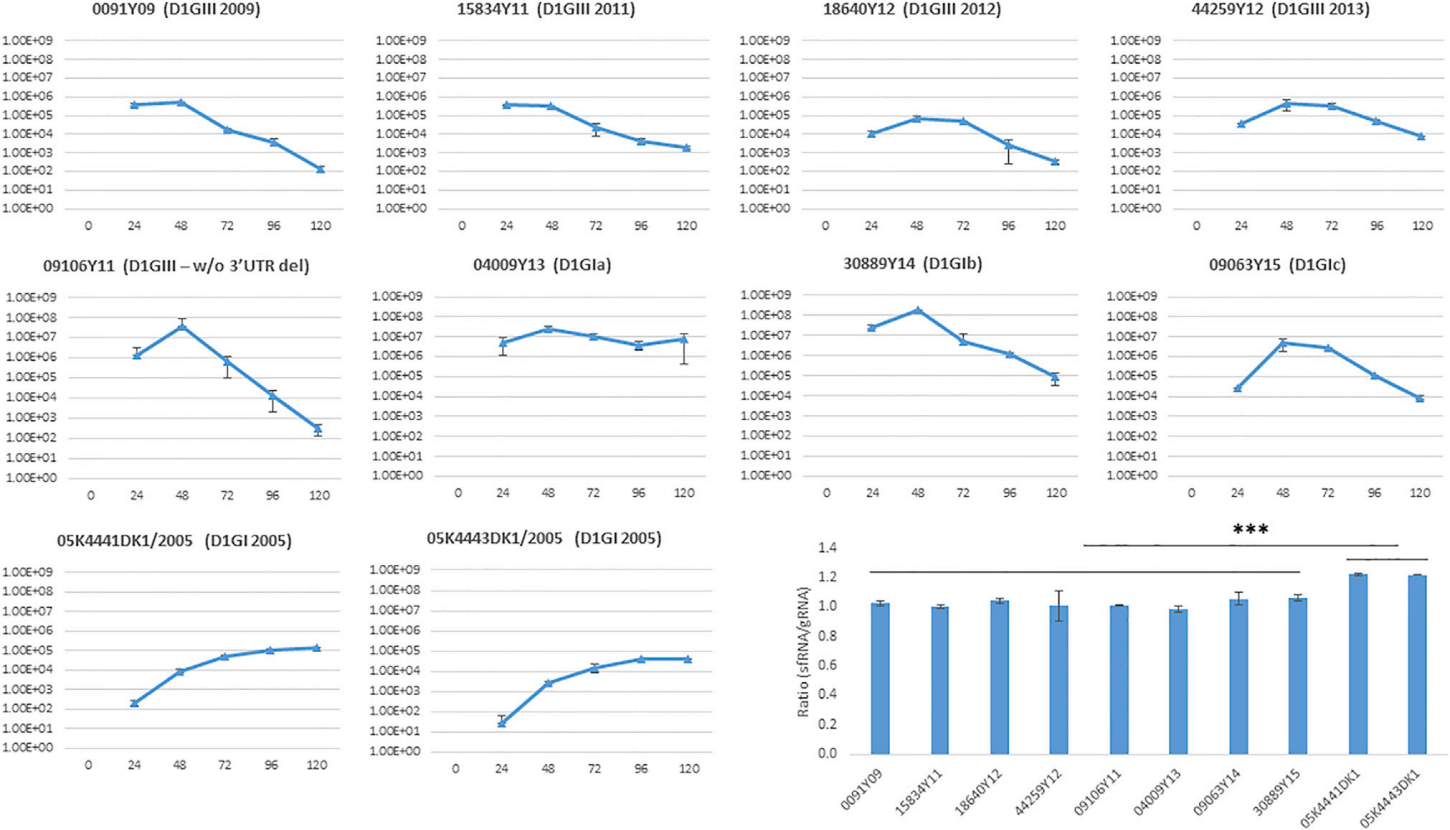
Replication Kinetics and Viral RNA Copy Fluctuations (gRNA and sfRNA) of DENV-1 Lineages and Strains in Huh-7 Mammalian Cells *In vitro* experiments were conducted at an MOI of 0.1. The y axis represents virus titers (pfu/mL), whereas the x axis refers to hours post infection (hpi). The bar chart shows the sfRNA:gRNA ratio for each isolate at 24 hpi. SG(EHI)D1/0091Y09, SG(EHI)D1/15834Y11, SG(EHI)D1/18640Y12, and SG(EHI)D1/44259Y12 isolates possess the 21-nucleotide deletion in the hypervariable region of the 3′ UTR (please see also Figure S2). The deletion is absent in SG(EHI)D1/09106Y11; SG(EHI)D1/04009Y13; SG(EHI)D1/30889Y14; SG(EHI)D1/09063Y15; 05K4441DK1/2005; and 05K4443DK1/2005 isolates. D1, DENV-1; GI, genotype I; GIII, genotype III; w/o, without; del, deletion. ***p ≤ 0. 001.

**Figure 7:**
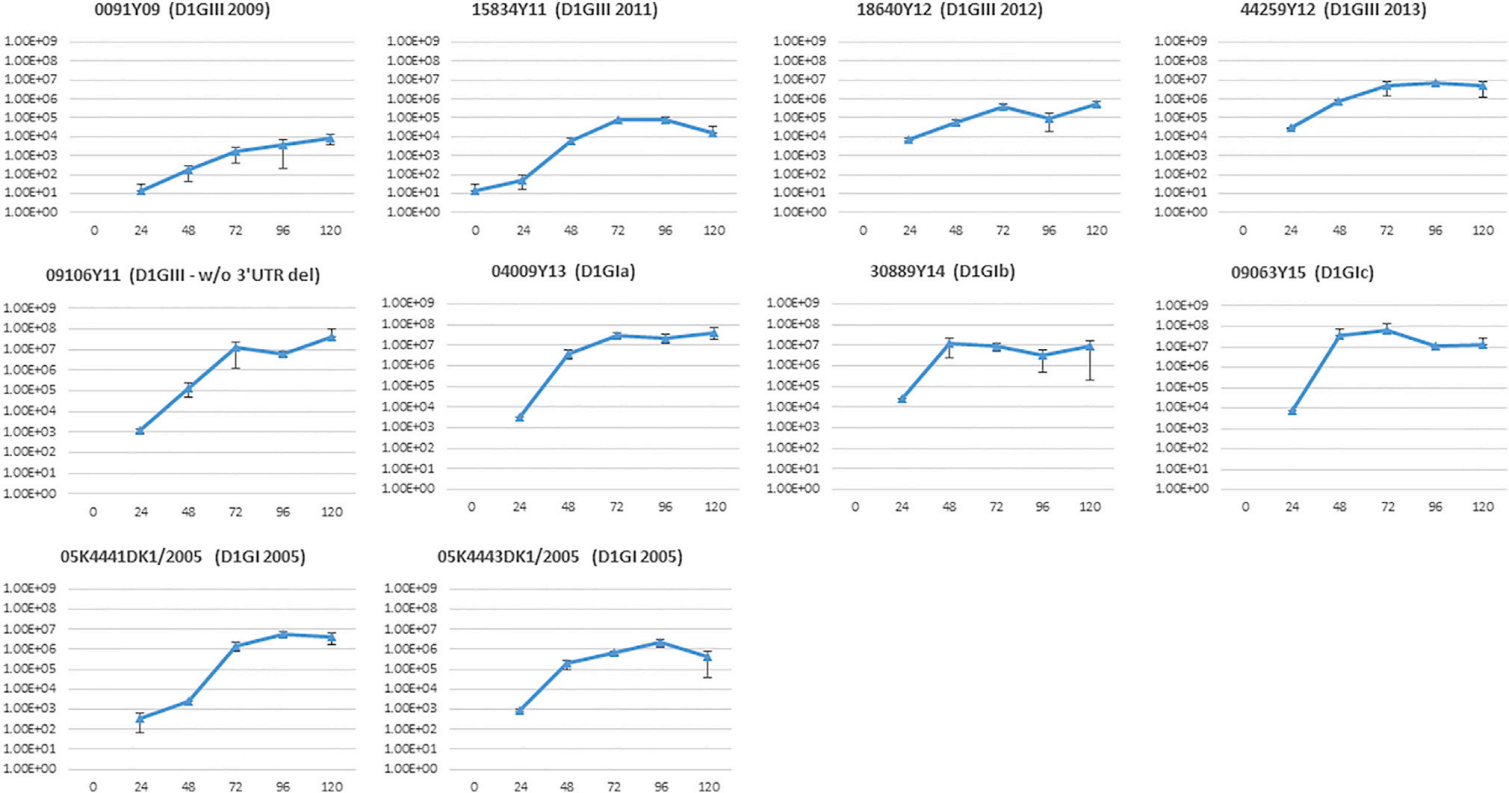
Replication Kinetics and Viral RNA Copy Fluctuations (gRNA and sfRNA) of DENV-1 Lineages and Strains in C6/36 *Aedes albopictus* Cells *In vitro* experiments were conducted at an MOI of 0.1. The y-axis represents virus titers (pfu/mL) whereas the x axis refers to hours post infection (hpi). The bar chart shows the sfRNA:gRNA ratio for each isolate at 24 hpi. SG(EHI)D1/0091Y09, SG(EHI)D1/15834Y11, SG(EHI)D1/18640Y12, and SG(EHI)D1/44259Y12 isolates possess the 21-nucleotide deletion in the hypervariable region of the 3′ UTR (please see also Figure S2). The deletion is absent in SG(EHI)D1/09106Y11; SG(EHI)D1/04009Y13; SG(EHI)D1/30889Y14; SG(EHI)D1/09063Y15; 05K4441DK1/2005; and 05K4443DK1/2005 isolates. D1, DENV-1; GI, genotype I; GIII, genotype III; w/o, without; del, deletion.

Having seen indifferent replication profiles in mosquito cells, we were first intrigued to investigate whether the 21-nucleotide deletion in the 3′ UTR HVR influenced the replication ability of genotype III lineages in mammalian cells. HVR is located at the first 45 nucleotides of the 3′ UTR of DENV-1. The region accommodates secondary stem-loop structure required for the virus interaction with cellular and viral proteins during the replication process (Dash et al., 2015). Previous studies have proposed that the complete, but not partial, HVR is essential for efficient replication of DENV-1 in mammalian cells (Tajima et al., 2007). A partial deletion of 19 nucleotides (from 15 to 33 nucleotides of HVR) exhibits a negligible effect on the replication of DENV-1 *in vitro* (Tajima et al., 2006). The secondary structure prediction analysis from a recent study demonstrated that the 21-nucleotide deletion does not alter the overall stem-loop structure of the 3′ UTR, suggesting that this partial deletion is unlikely to affect the replication ability of DENV-1. To further confirm this, we compared the replication profiles of genotype III strains with [genotype III 2011, 2012, 2013, and SG(EHI)D1/0091Y09] and without [SG(EHI)D1/09106Y11] the 21-nucleotide deletion in Huh-7 cells. Our analysis showed that the replication patterns were not significantly different between the two groups, suggesting that the deletion does not substantially affect the replication ability of DENV-1 in both mammalian (Figure 6) and mosquito cells (Figure 7).

Then, we compared the replication profiles between genotype I and III strains in mammalian cells. All viruses, except D1/SG/05K4441DK1/2005 and D1/SG/05K4443DK1/2005 of the 2005 epidemic lineage, demonstrated similar replication profiles in Huh-7 cells. They achieved the peak titers at 48 hours post infection (hpi), whereas D1/SG/05K4441DK1/2005 and D1/SG/05K4443DK1/2005 reached the peak titers at 120 hpi. Interestingly, genotype I lineages circulated after 2011 (Ia, Ib, and Ic) and SG(EHI)D1/09106Y11 strain achieved significantly higher peak titers than genotype III strains (t test; p value < 0.0001), albeit their relatively low transmission success when compared with genotype III lineages, especially the 2013 epidemic lineage. Moreover, both 2005 genotype I epidemic isolates and genotype Ia sustained virus titers longer than that of the remaining virus strains, in which the titers dropped quickly after 48–72 hpi (Figure 6). Because the number of host cells was comparable at an equal MOI among all virus strains, we postulated that the sustainability of live virus titers in mammalian cells might be explained, at least in part, by the potential ability of respective virus strains to evade the host innate immune response.

**Figure 8:**
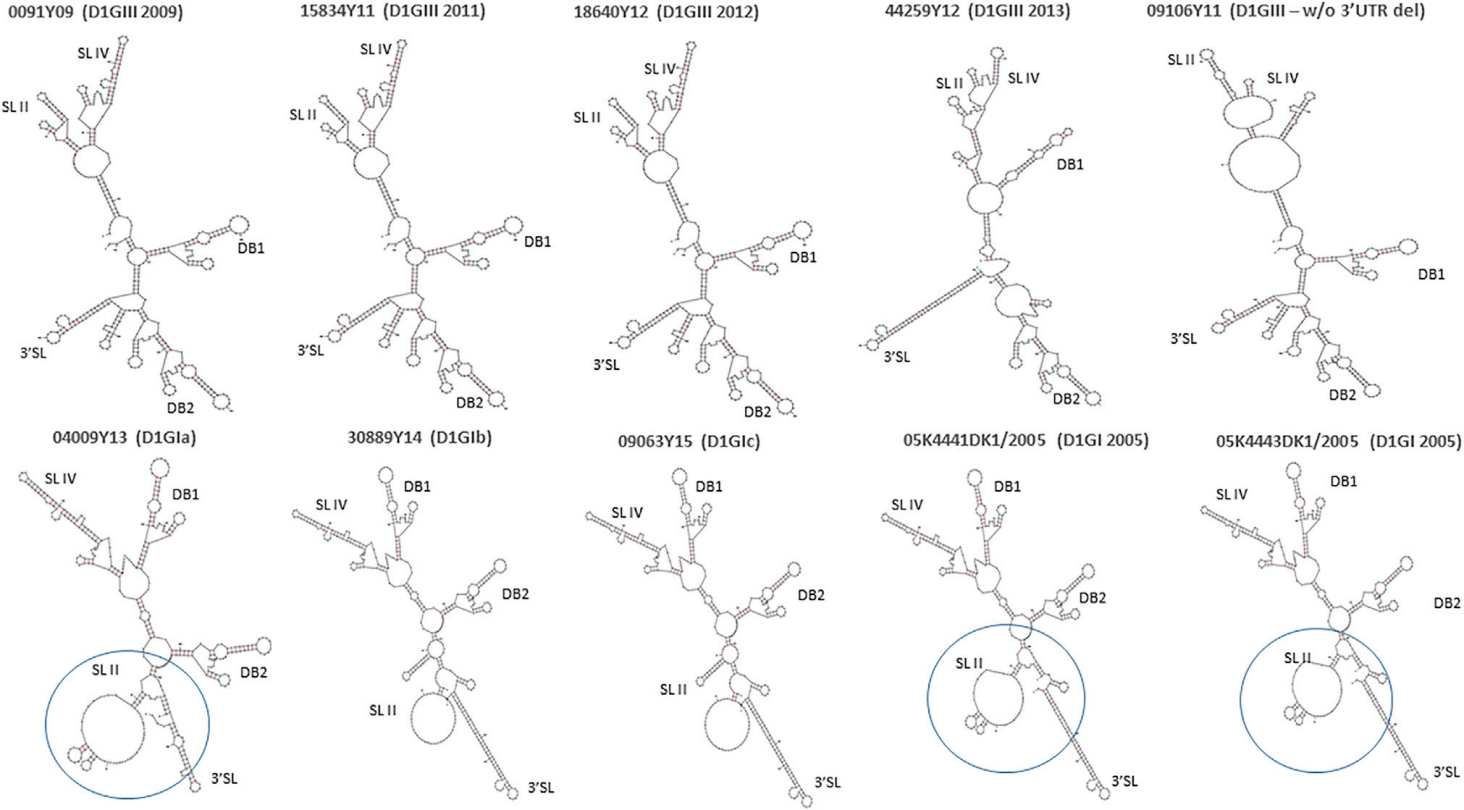
3′ UTR Structure of DENV-1 Strains The secondary structure of DENV-1 3′ UTR were predicted using the mfold web server under standard conditions (37°C). The analysis was performed by using complete 3′ UTR sequences. SG(EHI)D1/0091Y09; SG(EHI)D1/15834Y11; SG(EHI)D1/18640Y12; and SG(EHI)D1/44259Y12 isolates possess the 21-nucleotide deletion in the hypervariable region of the 3′ UTR (please see also Figure S2). The deletion is absent in SG(EHI)D1/09106Y11; SG(EHI)D1/04009Y13; SG(EHI)D1/30889Y14; SG(EHI)D1/09063Y15; 05K4441DK1/2005; and 05K4443DK1/2005 isolates. The circles represent SLII structure of 2005 genotype I epidemic strains and genotype Ia. D1, DENV-1; GI, genotype I; GIII, genotype III; SL, stem loop; DB, dumbbell. See also Figure S3.

It has been proposed previously that excess production of sub-genomic RNA (sfRNA) relative to genomic RNA (gRNA) and sequence variations within the sfRNA region attenuate type I interferon response, and thereby enhance the epidemiological fitness of DENV-2 (Manokaran et al., 2015). Therefore, we investigated the sequence and sfRNA:gRNA copy number variations between genotype I and III strains. The secondary structure prediction based on the full-length DENV-1 3′ UTR revealed that stem loop II (SL II) structures of 2005 genotype I epidemic strains and genotype Ia were similar, but different from other genotype I (Ib and Ic) and genotype III strains (Figure 8). SL II is important for the production of sfRNA (Pijlman et al., 2008), and thus warrants further studies to determine any phenotypic role in observed structural differences. However, the pseudoknot interactions that influence the efficiency of cellular 5′-3′ exoribonuclease and thus modulate the viral ability to generate nuclease-resistant sfRNA (Funk et al., 2010) remained unchanged in all strains (see Figure S3).

Then, we compared the sfRNA:gRNA copy number ratio between genotype I and III strains, obtained by using a newly developed in-house qPCR assay. Interestingly, 2005 genotype I epidemic strains, but not genotype Ia, produced significantly higher sfRNA:gRNA ratios at 24 hpi (Wilcoxon rank-sum test; p value < 0.001). As postulated earlier for DENV-2 (Manokaran et al., 2015), relatively low gRNA produced by 2005 genotype I epidemic strains, especially at early time points, could dampen the interferon response, allowing the virus titers to sustain in mammalian cells (Figure 6). The extended period of live virus survival could have facilitated transmission and thereby, the epidemic potential. Indeed, both 2005 genotype I strains used in the analysis (D1/SG/05K4441DK1/2005 and D1/SG/05K4443DK1/2005) were epidemic strains (Schreiber et al., 2009). Our findings, therefore, suggest that the “one-two punch” phenomenon (Manokaran et al., 2015) may potentially hold true for other DENV serotypes as well. However, the ratio between sfRNA and gRNA of genotype III strains was not significantly different, despite genotype III 2013 lineage being the dominant strain during the 2013–14 epidemic (Hapuarachchi et al., 2016b), implying that the transmission success and epidemic potential of virus lineages is multi-factorial, and may not necessarily be explained by a single phenomenon. Instead, the likely explanation of episodic, epidemic-scale transmission of DENV-1 in Singapore is the reduction of herd immunity against DENV-1 over the decades of DENV-2 predominance. This notion is supported by a recent study that showed low DENV-1 seroprevalance rate (14.2%) among young adults in the country (Low et al., 2015).

In conclusion, although the fitness, evolution, and host adaptation are important determinants of epidemic potential of virus lineages, our findings suggest that non-viral factors also play a substantial role in the transmission success of lineages in heterogeneous virus populations. Notable examples of these non-viral factors are the host immune pressure (herd immunity) and vector abundance. Because we did not perform *in vivo* studies to determine any fitness advantage of “established” strains in local vectors, the probability of vector-virus adaptation cannot be ignored and warrants further studies. Although the monitoring of virus populations is instrumental in identifying the emergence and diffusion of lineages with epidemic potential, our observations emphasize that successful control of vector-borne diseases requires a holistic understanding of vector, human host, and viral factors that contribute to disease transmission.

## Methods

All methods can be found in the accompanying Transparent Methods supplemental file.

## Data and Software Availability

Sequences used in the phylogenetic analysis, including the representative whole genome sequences, were deposited in Genbank database under the accession numbers GQ357692, JF960211, JN544407, JN544409-JN544411, KJ806939, KJ806941, KJ806943-KJ806947, KJ806949-KJ806951, KJ806953, KJ806959, KJ806961, KJ806963, KR779783, KX224261, KX224263, MF033196-MF033261.

## Supplemental Information

Document S1. Transparent Methods, Figures S1–S3, and Tables S1–S3

Data S1. XML Script File for the Whole-Genome-Based Phylogenetic Analyses, Related to Figure 1, Tables 1 and 2

Data S2. XML Script File for the Envelope Gene-Based Discrete Phylogeography Analysis, Related to Figures 3 and S1 and Tables S1 and S2
