# Supplementary material for: Highly Selective Transmission Success of Dengue Virus Type 1 Lineages in a Dynamic Virus Population: An Evolutionary and Fitness Perspective
Source: iScience. 2018 Jul 18;6:38–51. doi: 10.1016/j.isci.2018.07.008 (PMC6137288; doi:10.1016/j.isci.2018.07.008)
Supplement: Document S1. Transparent Methods, Figures S1–S3, and Tables S1–S3 [file mmc1.pdf]

ISCI, Volume *6*

Supplemental Information

Highly Selective Transmission Success of Dengue Virus Type 1 Lineages in a Dynamic Virus Population: An Evolutionary and Fitness Perspective

Carmen Koo, Wei Ping Tien, Helen Xu, Janet Ong, Jayanthi Rajarethinam, Yee Ling Lai, Lee-Ching Ng, and Hapuarachchige Chanditha Hapuarachchi

Supplemental figures

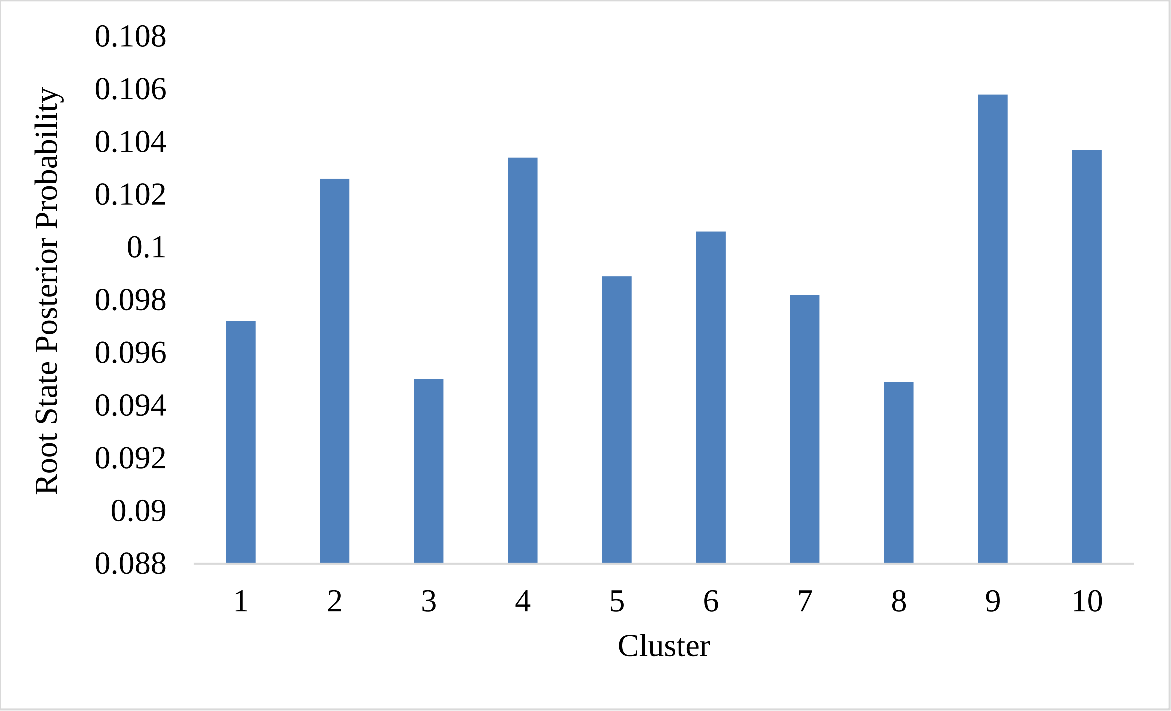

Figure S1. Related to Figure 3. Root state posterior probability of DENV-1.

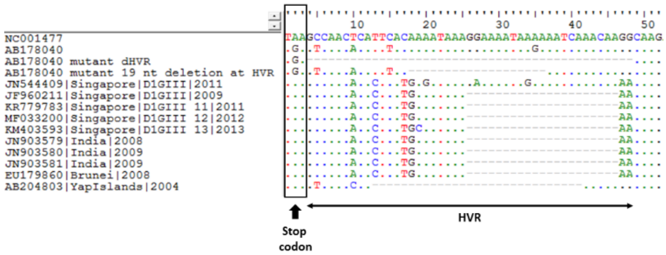

Figure S2. Related to Figures 6, 7 and 8. Schematic diagram of nucleotide deletions reported in the hypervariable region (HVR) of 3'UTR of DENV-1. Dashed lines represent nucleotide deletions. The figure includes global reference strains retrieved from Genbank database (NC001477, AB178040, JN903579, JN903580, JN903581, EU179860 and AB204803) for comparison. D1GIII: DENV-1 genotype III; D1GIII_11: DENV-1 genotype III 2011; D1GIII_12: DENV-1 genotype III 2012; D1GIII_13: DENV-1 genotype III 2013.

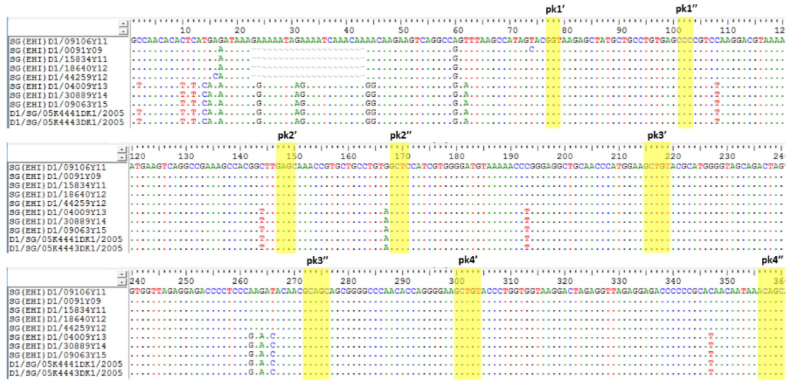

Figure S3. Related to Figure 8. Schematic diagram of putative pseudoknot-forming residues in 3'UTR of DENV-1. The diagram represents positions 1-360 after the stop codon. Regions highlighted in yellow refer to the predicted pseudoknot-forming sequences where base-pairing can occur to form the pseudoknot (pk) structures.

Supplemental tables

Table S1. Related to Figure 3. Bayes factor values of diffusion pathways.

No.	Clusters	Bayes factor value
1	between Cluster 3 and Cluster 9	368.4816194
2	between Cluster 2 and Cluster 4	322.0686685
3	between Cluster 3 and Cluster 4	286.9818883
4	between Cluster 4 and Cluster 9	224.2577204
5	between Cluster 1 and Cluster 2	186.974222
6	between Cluster 6 and Cluster 8	171.1916487
7	between Cluster 5 and Cluster 7	154.3323991
8	between Cluster 9 and Cluster 10	152.2193439
9	between Cluster 1 and Cluster 8	137.3399383
10	between Cluster 4 and Cluster 10	115.5293902
11	between Cluster 7 and Cluster 8	108.8632512
12	between Cluster 1 and Cluster 3	106.9446763
13	between Cluster 7 and Cluster 10	101.2540419
14	between Cluster 6 and Cluster 7	93.93485338
15	between Cluster 4 and Cluster 7	93.69136385
16	between Cluster 3 and Cluster 6	91.14921522
17	between Cluster 7 and Cluster 9	81.51131661
18	between Cluster 3 and Cluster 10	78.51693016
19	between Cluster 3 and Cluster 7	74.8352818
20	between Cluster 6 and Cluster 9	74.71606689
21	between Cluster 2 and Cluster 6	70.73170263
22	between Cluster 5 and Cluster 9	66.26096191
23	between Cluster 8 and Cluster 10	63.24082002
24	between Cluster 1 and Cluster 10	59.92942675

25	between Cluster 1 and Cluster 6	58.75353967
26	between Cluster 2 and Cluster 5	58.6198634
27	between Cluster 2 and Cluster 7	53.4630253
28	between Cluster 5 and Cluster 8	50.5958405
29	between Cluster 6 and Cluster 10	47.00862332
30	between Cluster 5 and Cluster 10	46.06235802
31	between Cluster 4 and Cluster 5	24.04903629
32	between Cluster 1 and Cluster 9	23.84063346
33	between Cluster 1 and Cluster 4	19.53090586
34	between Cluster 4 and Cluster 6	14.80692466
35	between Cluster 3 and Cluster 5	12.90426014

Table shows only the diffusion links with Bayes factor values greater than 3.

Table S2. Related to Figure 3. Summary of the most probable location of origin for each established strain of DENV-1.

DENV-1 strains	Most probable local origin	Root State Posterior Probability	Location of first reported sequence
Genotype III 2011	Cluster 4	0.2097	Cluster 1
**Genotype III 2012**	**Cluster 6**	**0.544**	**Cluster 6**
Genotype III 2013	Cluster 6	0.1766	Cluster 4
Genotype Ia 2013	Cluster 6	0.1445	Cluster 9
**Genotype Ib 2014**	**Cluster 4**	**0.2745**	**Cluster 4**
**Genotype Ic 2015**	**Cluster 4**	**0.6459**	**Cluster 4**

DENV-1 strains, of which the most probable root state matches to the location of its first detected sequence, are shown in bold.

Table S3. Related to Table 3. Neutral sites among the non-synonymous substitutions detected in six "established" lineages of DENV-1

Substitution[¶]	Lineage	Selection Pressure analysis[£]				
		SLAC	FEL	IFEL	FUBAR	MEME
prM-I120M	GIII 2011; DIII 2012	Neu	Neu	Neu	Neg (0.99)	Neu
prM-V135A	GIb	Neu	Neu	Neu	Neg (0.99)	Neu
E-I129T	GIII 2011; GIII 2012	Neu	Neu	Neu	Neg (0.93)	Neu
E-T359S	GIb	Neu	Neu	Neu	Neu	Epi (0.04)
E-S449N	GIII 2012	Neu	Neu	Neu	Neg (0.98)	Neu
NS1-D278N	GIII 2011; GIII 2013	Neu	Neu	Neu	Neu	Neu
NS2A-S142P	GIII 2012	Neu	Neu	Neu	Neu	Neu
NS2A-M168I	GIa	Neu	Neu	Neu	Neu	Neu
NS5-I114V	GIa	Neu	Neu	Neu	Neg (0.94)	Neu
NS5-P137H	GIb	Neu	Neu	Neu	Neg (0.98)	Neu
NS5-I181V	GIb	Neu	Neu	Neu	Neu	Neu
NS5-V413I	GIb; GIc	Neu	Neu	Neu	Neu	Neu
NS5-P525S	GIa; GIb	Neu	Neg (0.03)	Neu	Neg (0.98)	Neu

NS5-D554E	GIa	Neu	Neu	Neu	Neu	Neu
NS5-L562Q	GIa	Neu	Neu	Neu	Neg (0.95)	Neu

[¶]NCBI reference genome NC_001477 was used as the wild type sequence
[£]The data is given for the residues identified in genetic signatures of each "established" lineage (Tables 1 and 2). The table includes only the neutral sites determined by at least three methods. The sites under purifying and positive selection are given in Table 3. P-values (SLAC, FEL, IFEL and MEME) and posterior probability (FUBAR) are given in brackets. E=envelope protein; Epi=episodic selection; GI=genotype I; GIII=genotype III; prM=Precursor membrane protein; Neg=purifying selection; Neu=neutral; NS=non-structural proteins; Pos=positive selection

Transparent Methods

Sample collection

Whole blood samples of dengue-suspected patients were received by the Environmental Health Institute (EHI) from an extensive network of hospitals and general practitioner clinics located throughout Singapore. The present study included samples received by EHI from 2011 to 2016.

Ethics statement

All DENV-positive sera used for the genome sequencing were collected after obtaining the written informed consent from respective patients. All sera were utilized in accordance with the guidelines approved by the Institutional Review Board of National Environmental Agency, Singapore (IRB003.1).

Diagnosis of Dengue virus infections

In Singapore, the detection of NS1 antigen is used as the primary criterion for the confirmation of acute dengue infections. In the present study, Dengue virus (DENV) infection was confirmed by using the SD Bioline Dengue Duo kit (Standard Diagnostics INC., South Korea). SD Bioline Dengue Duo kit is a one-step rapid diagnostic test designed to detect both NS1 antigen and antibodies to DENV (Dengue IgG/IgM) in human serum, plasma or whole blood. The kit has demonstrated 93.9% sensitivity and 92.0% specificity in local cohorts (Gan et al., 2014).

Serotyping of DENV

The serotype of DENV in each NS1 positive sample was determined by using a protocol described elsewhere (Lai et al., 2007). DENV RNA was extracted from 140 µl of each sample serum by using the QIAamp Viral RNA Mini Kit (QIAGEN, Hilden, Germany), according to the manufacturer's guidelines. Forty units of RNaseOUT, a ribonuclease inhibitor (Life Technologies Corporation, USA), were added to each RNA elute to minimize degradation. Real-time reverse transcription polymerase chain reaction (rRT-PCR) was carried out in LightCycler 2.0 (Roche Diagnostics GmbH, Mannheim, Germany), by using the LightCycler RNA Master Hyb Probe Kit (Roche Diagnostics GmbH, Mannheim, Germany). The10 µl reaction mixture included 1X LightCycler RNA Master Hyb Probe mix, 3.25 mM of $Mn(Oac)_2$, 0.3 µM of each primer, 0.3 µM of DENV-1 probe, 0.15 µM of DENV-2 probe, 0.12 µM of DENV-3 probe and 0.2 µM of DENV-4 probe. The primer and probe sequences are available elsewhere (Lai et al., 2007). All reactions included 1 µl of RNA template, except for the negative controls, in which

RNA was replaced with molecular grade water. The single cycle reverse transcription step was carried out at 61^0C for 20 min, followed by 95^0C for 1 min. The amplification cycle included 45 cycles of denaturation at 95^0C for 1 sec, annealing at 59^0C for 15 sec and extension at 72^0C for 10 sec. The melting curves were acquired through a single cycle of 95^0C for 30 sec, followed by a continuous acquisition mode from 95^0C to 40^0C at a slope of 0.1^0C per sec. The melting curves and amplification curves were obtained by using the LightCycler Data Analysis Software version 4.05 (Roche Diagnostics GmbH, Mannheim, Germany).

Isolation of DENV for genome sequencing

DENV was isolated from sera using the *Ae. albopictus* C6/36 mosquito cell line (ATCC CRL-1660). Briefly, the monolayer of C6/36 cells at ~80% confluence was inoculated with 50 µl of serum in L-15 maintenance medium containing 3 % FCS at 33°C to allow for virus adsorption and replication. The infected fluid was harvested after 5-10 days. The presence of DENV in cell supernatants was confirmed by an immunofluoresent assay (IFA), during which cells were reacted with either DENV group-specific or serotype-specific monoclonal antibodies derived from hybridoma cultures (ATCC HB-46, HB-47, HB-48, and HB-49). The fluorescein isothiocyanate-conjugated goat anti-mouse antibody was used as the detector. Based on the original virus load, a maximum of three passages was done for each sample to minimize the cell-culture adaptive mutations.

Sequencing of envelope gene and whole genome of DENV

Envelope *(E)* gene sequences were generated directly from patient sera and the whole genomes were generated from isolates obtained from selected patient sera. Samples for whole genome sequencing were selected to represent different groups of viruses identified based on *E* gene phylogeny to cover observed genetic diversity of local DENV-1 population. For each virus group, genomes of at least two isolates detected throughout their transmission period, particularly during the early and late phases of their presence in the same locality, were fully sequenced. The number of isolates selected from each group was based on the total number of *E* gene sequences available for respective groups.

Complementary DNA (cDNA) was synthesised from extracted RNA by using ProtoScript II First Strand cDNA Synthesis system (New England Biolabs, Massachusetts, USA) according to the recommended protocol. The complete *E* gene (~1.5 kb) and complete genome were amplified by PCR using 0.5 µM of DENV serotype-specific primers and 1X PhusionTM Flash High-Fidelity PCR Master Mix (Finnzymes, Lafayette, CO). The amplification protocol for both *E* gene and the complete genome was as follows: initial denaturation at 98°C for 5 sec, 35 cycles of denaturation at 98°C for 5 sec, annealing at 60°C for 8 sec, extension at 72°C for 25 sec and final extension at 72°C for 1 min. The primers used for the amplifications have previously been described (Koo et al., 2013). Amplified products were purified using GeneJET PCR purification kit (Thermo Scientific, Massachusetts, USA) according to manufacturer's instructions. Sequencing of purified PCR products was performed at a commercial facility according to the BigDye Terminator Cycle Sequencing kit (Applied Biosystems, USA) protocol.

Assembly and analysis of genome sequences

Raw nucleotide sequences were assembled using the Lasergene package version 8.0 (DNASTAR Inc., Madison, WI, USA). Contiguous sequences were aligned using BioEdit 7.0.5 software

suite(Hall, 1999). Whole genome (n=239) and *E* gene (n=792) sequences analysed in the present study were compared with whole genome sequences (n=1768) retrieved from the GenBank database in BioEdit 7.0.5 software suite (Hall, 1999) to identify unique substitutions and genetic signatures of study isolates.

Data and software availability

Sequences used in the phylogenetic analysis, including the representative whole genome sequences, were deposited in Genbank database under the accession numbers GQ357692, JF960211, JN544407, JN544409-JN544411, KJ806939, KJ806941, KJ806943-KJ806947, KJ806949-KJ806951, KJ806953, KJ806959, KJ806961, KJ806963, KR779783, KX224261, KX224263, MF033196-MF033261.

Definition of a strain

DENV is composed of four serotypes and each serotype includes multiple genotypes. Each genotype is a broad classification to allow genetically diverse strains from different geographical regions to be clustered together. The empirical evidence shows that the envelope gene nucleotide divergence cut-off for DENV-1 strains of different genotypes ranges from 1.6% to 5.6% (Goncalvez et al., 2002). In a recent study, we demonstrated that DENV-1 epidemic strains are composed of multiple variants that show less than 1% nucleotide divergence (Hapuarachchi et al., 2016). Therefore, a strain was defined based on >= 1% nucleotide divergence and at least 80% bootstrap support in the *E* gene phylogeny. Besides the % nucleotide distance, 80% bootstrap support was considered to provide phylogenetic robustness of any defined strain.

Phylogenetic analysis of complete polyprotein sequences

A maximum clade credibility (MCC) tree was constructed using Markov Chain Monte Carlo (MCMC) method available in the Bayesian Evolutionary Analysis by Sampling Trees (BEAST) software package v1.7.4 (Drummond and Rambaut, 2007). The dataset included the representative DENV-1 whole polyprotein sequences of 93 study isolates and 94 sequences retrieved from the GenBank database. Out of 239 whole genome sequences, a subset of sequences (n=93) was selected to represent each group of viruses included in the whole genome sequencing. This subset included sequences reported during different time periods to capture the temporal variations, regardless of the locality, because the geography was not a consideration of the phylogenetic analysis. This “subset” selection was done to minimize the overcrowding of phylogeny.

The general time reversible model with gamma distribution and invariant sites (GTR+Γ_5+ I) was selected as the best fitting substitution model based on the jModelTest (Darriba et al., 2012) analysis. In order not to assume any particular demographic scenario as a priori, a relaxed uncorrelated lognormal clock (Drummond et al., 2006) and the Bayesian skyline plot (BSP) coalescent model (Lemey et al., 2009) was used to estimate the most probable origin, nucleotide substitution rate and the time to most recent common ancestor (tMRCA) of different lineages. Because the empirical evidence suggests that DENV-1 lineages evolve at different rates (Goncalvez et al., 2002), a strict clock was not preferred. The prior for the substitution rate was set at default settings to allow estimation of the rates among tree branches (Pls see supplemental Data File S1 for prior information). The MCMC chain was run for 200 million generations

sampling every 20,000 states. The effective sampling size (ESS) values of more than 200 were considered as a sufficient level of sampling. The posterior tree distribution was summarized using TreeAnnotator v.1.7.4 program (Drummond and Rambaut, 2007), with 10% burn-in and the final MCC tree was visualized using FigTree v.1.4.3 (http://tree.bio.ed.ac.uk/software/figtree/).

Assigning of locations to each sequence

In Singapore, dengue control is a joint effort between the National Environment Agency (NEA) and the Ministry of Health (MOH). The effort is backed up by a strong surveillance framework that includes cases, virus and vectors. Cases are reported through a mandatory notification system. All cases are laboratory confirmed. Once a case is confirmed, MOH conducts an epidemiological investigation that includes interviewing patients whenever possible to determine whether a case is imported or locally acquired. If locally acquired, MOH provides the postal code information of the most probable location of infection for each case. This could be the residential, workplace or any other address. In the present study, we used location data assigned by MOH to each case. The arbovirus surveillance programme uses this data to monitor the spatial distribution of virus strains on a weekly basis, with a view of mapping those that establish autochthonous transmission. NEA uses this information to define dengue disease clusters and thereby to identify hotspots of DENV transmission.

Phylogeography analysis of envelope gene sequences

Partitioning Around Mediods (PAM) was used to geocluster virus data into distinct clusters based on postal code information assigned to each sequence. PAM is a partitioning technique of clustering that minimizes the distance between a particular point in a cluster and the point designated as the centre of the cluster. The average distance to the next nearest postal code (18 m for the analysed dataset) was calculated by using the "calculate distance band from neighbour count" tool in ArcGIS 10.5. Using the geographical distance of cases as the dissimilarity measure, the respective virus sequences were grouped into 10 clusters. The average distance between two clusters was 5,250 m (Range: 2,986-6,027 m). The optimal number of clusters (k=10) was determined by comparing the Silhouette width of different cluster numbers.

The complete *E* gene sequences of 792 randomly selected DENV-1 strains from 10 defined geographical locations were used for phylogeography analysis. At least one representative was chosen from a group of identical sequences from each location per epidemiological week (EW). jModelTest (Darriba et al., 2012) suggested the Tamura-Nei model with gamma rate categories (TN93+Γ_5) as the best-fitting substitution model. The analyses were performed using a flexible Bayesian skyline demographic prior and a relaxed uncorrelated lognormal clock (Drummond et al., 2006). A Bayesian Stochastic Search Variable Selection (BSSVS) procedure was used to identify significant diffusion pathways among 10 clusters included in the discrete phylogeographic analysis (Lemey et al., 2009) (Pls see supplemental Data File S2 for prior information). Four MCMC analyses for 100 million generations each, sampling every 10,000 states, were combined in LogCombiner (http://tree.bio.ed.ac.uk/software), after removing 10% burn-in. SPREAD 1.0.4 (Bielejec et al., 2011) was used to analyse phylogeographic reconstructions resulting from Bayesian inference of spatiotemporal diffusion of tests established. Bayes factor (BF) values were calculated by comparing the posterior and prior probability of the individual rates in order to test the significant linkage between locations. A

BF>3 was considered well-supported, with classifications of substantial (BF>3), strong (BF>10), very strong (BF>30) and decisive (BF>100) (Faria et al., 2013; Lemey et al., 2009). The diffusion links between locations were visualized in Google Earth.

Selection pressure analysis

The selection pressure acting on each codon of whole genomes was measured by the ratio of non-synonymous to synonymous rates (dN/dS) computed in HyPhy open-source software package as implemented in Datamonkey web-server (Delport et al., 2010). Single likelihood ancestor counting (SLAC), fixed effects likelihood (FEL), internal fixed effects likelihood (IFEL), mixed effect model of evolution (MEME) and fast unbiased Bayesian approximation (FUBAR) methods were used to estimate the sites under selection. The GTR nucleotide substitution bias model (REV) and neighbour joining phylogeny were used as analytical parameters in different approaches. An integrative approach recommended previously (Delport et al., 2010) was employed in ascertaining the positive or negative selection at each site. The sites which were found to be non-neutral and be positively or negatively selected by all methods, and by at least three methods at significant levels were considered to be positively/negatively selected sites. The p-values = < 0.05 were considered as significant for SLAC, FEL, IFEL and MEME methods, whereas the posterior probability value of = > 0.9 was used for FUBAR.

Secondary structure analysis of 3'untranslated region

The secondary structures of DENV-1 3' untranslated region (UTR) of different lineages were predicted using the mfold web server (Zuker, 2003) at http://mfold.rna.albany.edu/?q=mfold under standard conditions (37^0C). The analysis included the complete sequences of 3' UTR.

***In vitro* replication kinetics**

Virus stock preparation

We determined the replication kinetics of 10 DENV-1 strains of genotypes I and III. They included six strains that established sustained transmission during the study period (Genotype III 2011; SG(EHI)D1/15834Y11; NCBI accession no. JN544407, Genotype III 2012; SG(EHI)D1/18640Y12; NCBI accession no. MG097875, Genotype III 2013; SG(EHI)D1/44259Y12; NCBI accession no. KM403575, Genotype Ia; SG(EHI)D1/04009Y13; NCBI accession no. KJ806953, Genotype Ib; SG(EHI)D1/30889Y14; NCBI accession no. MG097876, Genotype Ic; SG(EHI)D1/09063Y15; NCBI accession no. MF033232), two genotype III isolates reported in isolated cases in 2009 (SG(EHI)D1/0091Y09; NCBI accession no. JF960211) and 2011 (SG(EHI)D1/09106Y11; NCBI accession no. JN544409) as well as two genotype I isolates reported during the epidemic in 2005 (D1/SG/05K4441DK1/2005; NCBI accession no. EU081266 and D1/SG/05K4443DK1/2005; NCBI accession no. EU081267). All isolates were scaled-up up to four passages by using 25 µl of stock solution in *Aedes albopictus* C6/36 cell line (ATCC CRL 1660) as described in the virus isolation for genome sequencing section, to obtain a virus titre of at least 10^5 plaque-forming units (PFU) per ml. Virus stocks were kept at -80°C until further use.

Replication kinetics assay

Hepatoma cell line Huh-7 (Courtesy of Prof. Justin Chu, National University of Singapore) and *Aedes albopictus* C6/36 cell line (ATCC CRL 1660) were used to determine the replication kinetics of DENV-1 lineages in mammalian and mosquito cells respectively. These two cell lines have commonly been used by previous similar studies (Manokaran et al., 2015; Tajima et al., 2006). Huh-7 cells were scaled-up in Dulbecco's modified Eagle's medium (DMEM, Biopolis Shared Facilities, A*Star, Biopolis, Singapore) at 37°C with 5% CO2. The media contained 10% heat-inactivated Foetal Bovine Serum (FBS), 1% L-glutamine solution, 100 mM Penicillin/Streptomycin, 7.5% sodium bicarbonate and 10 mM HEPES. *Aedes albopictus* cell line (C6/36, ATCC CRL 1660) was scaled-up at 28°C in Leibowitch L-15 medium (Invitrogen Corp., Carlsbad, CA, USA), supplemented with 10% FBS, 1% L-glutamine solution and 100 mM Penicillin/Streptomycin. Maintenance medium for each cell line was similarly prepared, but supplemented with 3% FBS.

Huh-7 and C6/36 cells were seeded at ~2 x 10^5 cells in 24 well plates to approximately 85-90% confluence, before infecting with DENV-1 strains at multiplicity of infection (MOI) of 0.1 in duplicates. After one-hour adsorption at 37˚C, each virus suspension was removed and cells were washed twice with phosphate buffered saline (PBS). The cells were then incubated in maintenance media at 37°C for up to five days. Supernatants were harvested at 0 hr and every 24 hrs subsequently up to 120 hrs (0 to 5 days) post infection (hpi) and infectious viral titres were determined by plaque assay.

Quantification of infectious virus particles by plaque assay

Baby Hamster Kidney cells (BHK-21, courtesy of Novartis Institute of Tropical Diseases) were used to perform the plaque assay (Manokaran et al., 2015; Tan et al., 2013). BHK-21 cells were scaled-up in RPMI medium (Sigma-Aldrich Corp., St. Louis, MO, USA) at 37°C with 5% CO2. The plaque assay for the quantification of infectious virus titres was carried out for each virus as described elsewhere (Tan et al., 2013). In brief, BHK-21 cells were seeded in 24 well plates to obtain a monolayer of cells at 90-95% confluence. The viruses were serially diluted from 1:10^{-1} to 1:10^{-6} using serum-free culture media. Following one-hour adsorption, virus inoculum was discarded and 1.5% complete carboxymethyl cellulose (CMC) medium was added. At day 5, BHK-21 cells were fixed with 20 % formalin and stained with naphthol blue stain solution. Plaques were counted and expressed as PFU/ml.

Amplification of genomic and sub-genomic RNA

DENV-1 total RNA was extracted from infected cells using the QIAGEN QIAamp viral RNA mini kit (QIAGEN, Hilden, Germany) according to manufacturer's recommendations. DENV-1 genomic (gRNA) and sub-genomic (sfRNA) RNA levels were measured by using a SYBR green I-based one-step real-time quantitative RT-PCR assay carried out on a LightCycler® 2.0 (Roche Diagnostics GMbH, Mannheim, Germany). Primer gD1-F (5' AGCCATAGCACGGTAAGAGC 3') targeted the upstream (coding) region of DENV-1 3'UTR and captured gRNA and sfRNA. On the other hand, primer sfD1+3 (5' CCGTCCAAGGACGTAAAATG 3') was designed within the DENV-1 sfRNA region to capture any excess sfRNA production. We used a single reverse primer sfD1+3-R (5' CCCTCCCAAGACACAACGCAGC 3') that generated 211 bp (gRNA+sfRNA) and 174 bp (sfRNA) amplicons. The RT-PCR was performed by using QuantiTect SYBR Green RT-PCR Kit (Qiagen, Hilden, Germany) with 1 µl of RNA template and 1 µM of each primer in 10 µl reactions. The thermal profile of RT-PCR was 60°C for 10 min

and 95°C for 0 s, followed by 35 cycles of 94°C for 0 s, 46-50°C for 3 s and 72°C for 10 s, melting at 70°C for 30 s and a cooling step of 40°C for 30 s.

Quantification of genomic and sub-genomic RNA copy numbers

The concentration of purified amplicons of gRNA and sfRNA was measured in a NanoDrop spectrophotometer (NanoDrop Technologies, Inc. DE) and were cloned into the vector at an insert: vector ratio of 3:1 by using the NEB PCR cloning kit (New England Biolabs, USA) according to manufacturer's instructions. The presence of cloned inserts was confirmed by colony PCR. The plasmid minipreps were prepared by using the Wizard plus SV minipreps DNA purification kit (Promega Corporation, Madison, WI). The inserts were obtained after digestion with EcoRI (New England Biolabs, USA) and were gel purified by using the QIAquick Gel Purification kit (Qiagen, Hilden, Germany). The insert yield was quantified as above and copy numbers were calculated based on the product size and DNA concentration. The purified inserts were 10-fold serially diluted from 10^8 to 10^{-4} copies/ul and RT-PCR was performed for each dilution as described above. The standard curve, obtained by plotting quantification cycle values against known copy numbers for each dilution, was used to quantify the number of RNA copies in study samples. The ratio of sfRNA: gRNA copies was calculated to determine if any of the virus strains produced excess sfRNA.

Supplemental References

Bielejec, F., Rambaut, A., Suchard, M.A., and Lemey, P. (2011). SPREAD: spatial phylogenetic reconstruction of evolutionary dynamics. Bioinformatics *27*, 2910-2912.

Darriba, D., Taboada, G.L., Doallo, R., and Posada, D. (2012). jModelTest 2: more models, new heuristics and parallel computing. Nat Methods *9*, 772.

Delport, W., Poon, A.F., Frost, S.D., and Kosakovsky Pond, S.L. (2010). Datamonkey 2010: a suite of phylogenetic analysis tools for evolutionary biology. Bioinformatics *26*, 2455-2457.

Drummond, A.J., Ho, S.Y., Phillips, M.J., and Rambaut, A. (2006). Relaxed phylogenetics and dating with confidence. PLoS Biol *4*, e88.

Drummond, A.J., and Rambaut, A. (2007). BEAST: Bayesian evolutionary analysis by sampling trees. BMC Evol Biol *7*, 214.

Faria, N.R., Suchard, M.A., Rambaut, A., Streicker, D.G., and Lemey, P. (2013). Simultaneously reconstructing viral cross-species transmission history and identifying the underlying constraints. Philos Trans R Soc Lond B Biol Sci *368*, 20120196.

Gan, V.C., Tan, L.K., Lye, D.C., Pok, K.Y., Mok, S.Q., Chua, R.C., Leo, Y.S., and Ng, L.C. (2014). Diagnosing Dengue at the Point-of-Care: Utility of a Rapid Combined Diagnostic Kit in Singapore. PLoS One *9*, e90037.

Goncalvez, A.P., Escalante, A.A., Pujol, F.H., Ludert, J.E., Tovar, D., Salas, R.A., and Liprandi, F. (2002). Diversity and evolution of the envelope gene of dengue virus type 1. Virology *303*, 110-119.

Hall, T.A. (1999). BioEdit: a user-friendly biological sequence alignment editor and analysis program for Windows 95/98/NT. Nucl Acid Symp Ser *41*, 95-98.

Hapuarachchi, H.C., Koo, C., Kek, R., Xu, H., Lai, Y.L., Liu, L., Kok, S.Y., Shi, Y., Chuen, R.L., Lee, K.S., *et al.* (2016). Intra-epidemic evolutionary dynamics of a Dengue virus type 1 population reveal mutant spectra that correlate with disease transmission. Sci Rep *6*, 22592.

Koo, C., Nasir, A., Hapuarachchi, H.C., Lee, K.S., Hasan, Z., Ng, L.C., and Khan, E. (2013). Evolution and heterogeneity of multiple serotypes of Dengue virus in Pakistan, 2006-2011. Virol J *10*, 275.

Lai, Y.L., Chung, Y.K., Tan, H.C., Yap, H.F., Yap, G., Ooi, E.E., and Ng, L.C. (2007). Cost-effective real-time reverse transcriptase PCR (RT-PCR) to screen for Dengue virus followed by rapid single-tube multiplex RT-PCR for serotyping of the virus. J Clin Microbiol *45*, 935-941.
Lemey, P., Rambaut, A., Drummond, A.J., and Suchard, M.A. (2009). Bayesian phylogeography finds its roots. PLoS Comput Biol *5*, e1000520.
Manokaran, G., Finol, E., Wang, C., Gunaratne, J., Bahl, J., Ong, E.Z., Tan, H.C., Sessions, O.M., Ward, A.M., Gubler, D.J., *et al.* (2015). Dengue subgenomic RNA binds TRIM25 to inhibit interferon expression for epidemiological fitness. Science *350*, 217-221.
Tajima, S., Nukui, Y., Ito, M., Takasaki, T., and Kurane, I. (2006). Nineteen nucleotides in the variable region of 3' non-translated region are dispensable for the replication of dengue type 1 virus in vitro. Virus research *116*, 38-44.
Tan, L.K., Lam, S., Low, S.L., Tan, F.H., Ng, L.C., and Teo, D. (2013). Evaluation of Pathogen Reduction Systems to Inactivate Dengue and Chikungunya Viruses in Apheresis Platelets Suspended in Plasma. Advances in Infectious Diseases *Vol.03No.01*, 9.
Zuker, M. (2003). Mfold web server for nucleic acid folding and hybridization prediction. Nucleic Acids Res *31*, 3406-3415.
